# Effects of a 12-Minute Smartphone-Based Mindful Breathing Task on Heart Rate Variability for Students With Clinically Relevant Chronic Pain, Depression, and Anxiety: Protocol for a Randomized Controlled Trial

**DOI:** 10.2196/14119

**Published:** 2019-12-02

**Authors:** Muhammad Abid Azam, Vered Valeria Latman, Joel Katz

**Affiliations:** 1 York University Toronto, ON Canada

**Keywords:** mindfulness, mobile apps, heart rate variability, chronic pain, depression, anxiety

## Abstract

**Background:**

Mindfulness meditation (MM) is a commonly used psychological intervention for pain, mood, and anxiety conditions, but can be challenging to practice with severe symptoms without proper training. The Mindfulness Meditation app (MMA) is a supportive training tool specifically developed for this study to aid in the practice of mindful breathing using a smartphone.

**Objective:**

This study aims to evaluate the psychophysiological effects of the MMA. Specifically, the study will assess parasympathetic functioning using heart rate variability (HRV; primary outcome), pain and mood symptoms, mind-wandering and present moment awareness, and breath focus in groups of undergraduate participants who self-report clinically-relevant symptoms of chronic pain (CP) and depression or anxiety (DA) and condition-free (CF) participants who do not meet either criteria. We hypothesize that use of the MMA by study groups will lead to improved HRV, pain, and mood symptoms compared with groups who do not use the app.

**Methods:**

This study is a two-arm randomized controlled trial (RCT) recruiting through a Web-based research participation pool at York University in Toronto, Canada. We are aiming for minimum 60 participants in each of CP, DA, and CF groups. Upon arriving to the laboratory, participants will be prescreened for classification into groups of CP, DA, or CF. Groups will be randomly assigned by a 1:1 ratio to an MMA (MMA+) condition or MM condition without the app (MMA−) after a brief stress induction procedure. In MMA+, participants will practice mindful breathing with a smartphone and press breath or other buttons at the sound of audio tones if their awareness was on breathing or another experience, respectively. HRV and respiration data will be obtained during rest (5 min), stress induction (5 min), and meditation conditions (12 min). Participants will complete psychological self-report inventories before and after the stress induction and after the meditation condition. Separate linear mixed models will be used to examine HRV and self-report inventories comparing groups and treatment conditions.

**Results:**

Recruitment for the study began in November 2017 and is expected to be completed in winter of 2019-2020. As of July 2019, 189 participants have been recruited. The study’s main findings are expected to reveal a positive pattern of HRV responses in the CP, DA, and CF groups, such that a significant increase in HRV (*P*<.05) is detected in those randomized to the MMA+ condition in comparison with those randomized to the MMA− condition.

**Conclusions:**

This RCT will contribute to the burgeoning health psychology literature regarding the clinical relevance of HRV in assessment and treatment of psychological and medical conditions. Furthermore, possible ways to inform designs of MM training tools delivered by apps and Web platforms for CP, depression, and anxiety conditions’ treatment will be discussed.

**Trial Registration:**

Clinicaltrials.gov NCT03296007; https://clinicaltrials.gov/ct2/show/NCT03296007.

**International Registered Report Identifier (IRRID):**

DERR1-10.2196/14119

## Introduction

### Background

Mindfulness meditation (MM) is an element of the Buddhist traditions first introduced as a clinical intervention in western medicine by Kabat-Zinn [1]. Over the past 35 years, the practice has received significant interest in clinical and health psychology and, more recently, in neuro- and psychophysiology. A family of meditative practices based on mindfulness is commonly used as a psychological approach to mental illness and chronic pain (CP) management. Mindful breathing is central to the mindfulness practices and places an emphasis on paying nonjudgmental attention to one’s cognitive, emotional, and physical experiences while reorienting focus on breathing sensations to cultivate cognitive and emotion regulation and progressively relaxed states [2]. Mindfulness-based treatments have been shown to be effective in reducing symptoms of depression, anxiety, and chronic pain [3,4].

One proposed mechanism for mindful breathing benefits is the state relaxation effect mediated by parasympathetic vagus nerve activation because of its process of bringing awareness to breathing sensations while practicing acceptance of stressors such as worrying thoughts, negative emotions, and pain [5]. However, individuals with pain, depression, and anxiety disorders commonly share the physiological trait of a dysregulated autonomic nervous system (ANS), particularly with respect to parasympathetic stress recovery processes, which compromise vagal activation [6,7].

Rigid and inflexible types of mind wandering, such as worry and rumination, are typically seen in individuals with psychiatric illnesses [8,9] and chronic pain [10]. These stressors lead to prolonged physiological stress activation linked to health risks [6,11]. MM attempts to counteract unhealthy mind wandering by instilling present-oriented attention that is more conducive to appropriately rested and relaxed states in the absence of stressful demands [12]. Critical to the purported benefits of mindfulness is that the removal of stressful stimuli in the environment does not always coincide with a return to resting state. Physiological stress inevitably arises through pain, worry, rumination, and distressing emotions such as fear, anxiety, and anger. Patterns of worry and rumination are defined as *perseverative stressors*, as they are typically related to past or anticipated stressors [6]. Perseverative stress refers to persistent physiological stress that can linger for prolonged periods, sometimes subconsciously, creating an undertone of stress arousal and allostatic load associated with gradual and sustained withdrawal of vagal tone, quantifiable by low heart rate variability (HRV) [11]. Chronically low HRV is a sign that the ANS is *sympathetically dominant*, with persistent and excessive activation of the stress response that is less amenable to vagal-mediated parasympathetic recovery [6,11]. Mindfulness practice may be a key method to target the pathophysiological mechanism common to chronic pain, depression, and anxiety. Mindful breathing can enhance vagal activation through state relaxation effects, as multiple studies have shown that even brief mindfulness practice after stress significantly increases HRV [13,14]. This vagal-activating effect may have stress-relieving benefits for individuals with depression-anxiety symptoms [15]. With regards to pain, reductions in self-reported pain have been demonstrated in individuals practicing HRV-based biofeedback [16], and it has been theorized that increased vagal activation is connected to several analgesic mechanisms such as lowered systemic inflammation and inhibited pain-processing brain regions [17]. Thus, the vagal-activating treatment potential of a smartphone-based mindful breathing app may have clinical utility, given the strong research evidence associating low HRV and psychological [18] and pain conditions [7].

A potentially impeding factor for client populations in practicing mindful breathing is the demand characteristics of the silent, inwardly focused exercise that may naturally make them more saliently aware of their symptoms of depression (eg, rumination), anxiety (worry), and pain (sensations). Relatedly, a qualitative study has revealed experiential challenges to MM practice that must be addressed in clinical treatment contexts, including the fact that meditation is a difficult skill to learn and practice, and participants encounter difficult thoughts and feelings amidst practice [19]. This is all the more relevant to naïve meditators with pain, depression, and anxiety symptoms, making for a potentially challenging experience that discourages further MM practice.

Supportive training tools, such as smartphone apps, are widely used to receive audio guidance on MM, and evidence is emerging for their immediate and long-term effects on altering mood states [20]. However, few studies have investigated the clinical utility and physiological changes related to meditation-based smartphone apps [21]. Furthermore, audio instructions may not be readily applicable in participants with clinical symptoms as they may be more vulnerable to their mind-wandering effects amidst meditation practice. In 1 study, a computer-based mindful breathing task provided random audio tones during the practice to prompt participants to reorient attention to breathing sensations after pressing a button on the keyboard to indicate whether they had been paying attention to breathing or other experiences at the time of the tone [22]. The results showed higher instances of breath attention were correlated with increased HRV during the practice, suggesting a positive relaxation effect of the mindful breathing task. This paradigm could be effectively applied in a clinical context for individuals who may initially struggle to practice mindful breathing, such as those with chronic pain and severe depression-anxiety symptoms. Although multiple studies have shown that MM leads to HRV increases [13,22], there are no known studies that have investigated the effects of a smartphone-based mindful breathing task on HRV in individuals with chronic pain and severe depression-anxiety symptoms. One study provided naïve meditators training in the mindfulness technique of thought distancing using a smartphone app or traditional mindfulness practice without an app. They found significantly greater mindfulness and pleasantness and lower perceived difficulty associated with the smartphone app–based mindfulness practice compared with the nonapp conditions [23]. More investigations are needed to determine in what ways smartphone apps can be employed as an effective medium of delivering mindfulness training to vulnerable populations who are naïve to meditation.

A recent systematic review of mindfulness-related apps found over 500 apps in the marketplace, but only 23 were designed to provide mindfulness training, and they were largely focused on providing audiovisual guidance, timers, and reminders to practice [21]. The review revealed that only 1 randomized controlled trial (RCT) for a mindfulness app was underway at the time [24] and that, generally, there is limited evidence available for the efficacy of apps in increasing mindfulness [21]. Furthermore, the literature on existing mobile apps marketed for chronic pain reveals that, with few exceptions [25], they rarely adhere to scientific guidelines and that health care professionals are rarely involved in their development [26-29].

The aim of this study is to overcome some of the aforementioned limitations by designing an app with input from clinical researchers and empirically testing the app’s effectiveness in a sample of young adults with CP, anxiety, and depression. To cultivate mindfulness, a mobile-based Mindfulness Meditation app (MMA) was developed based on Burg et al’s [22] mindful breathing exercise that “assesses the participants’ ability to mindfully stay in contact with the bodily sense of the breath during an exercise aligned with breathing meditation” [22]. In lieu of verbal meditation instructions amidst practice, the task provides audio tones at random intervals to which practitioners respond by pressing keys to indicate *breath* (if they were attending to their breathing at the sound of the tone) or *other* (if they were attending to an experience other than the breath at the sound of the tone). This simplified task may be more suitable to beginner meditators and particularly those with various psychiatric and pain symptoms that make meditation practice more difficult [19]. In a previous pilot study of the MMA (VV Latman, MA, unpublished data, June 2017), participants with chronic pain and severe depression-anxiety symptoms randomized to the 12-min MMA task exhibited significant reductions in mood states of anxiety, anger, and overall distress.

**Figure 1:**
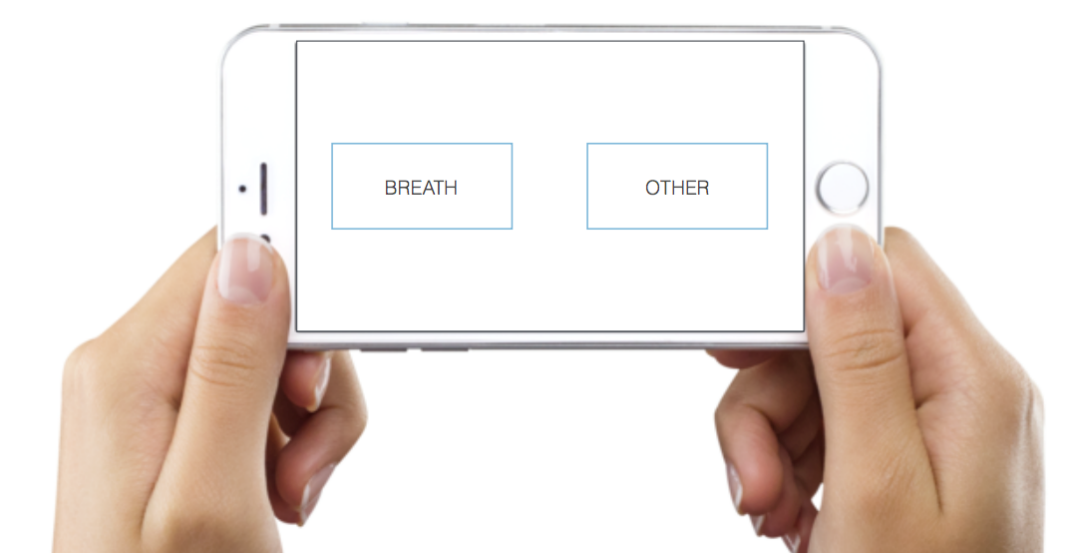
The Mindfulness Meditation App.

This study will evaluate the psychophysiological effects of a smartphone-based MMA (12 min; Figure 1) for individuals with clinically significant symptoms of major depression or anxiety (DA) or chronic pain. Specifically, the study aims to examine parasympathetic activity using HRV (primary outcome) in groups of participants who self-report clinically significant symptoms of DA and CP and condition-free (CF) participants who do not meet our criteria for either. In addition, given the linkages between HRV and the following psychological processes within psychological and medical conditions, the study will also assess mind wandering and present moment awareness, mood symptoms, and breath focus before and after the intervention. All study groups will be randomized to an MMA (MMA+) condition or a MM condition without the app (MMA−) after a brief stress induction procedure.

Overall, 3 major hypotheses (1a, 1b, 2a, 2b, and 3) will be tested.

### Primary End Point

#### Hypothesis 1a

HRV change scores during MM will be significantly higher in the CP and DA groups receiving MMA+ compared with corresponding CP and DA groups receiving MMA−, and will not differ between CF groups receiving MMA+ or MMA−.

#### Hypothesis 1b

HRV will significantly increase from the stress to MM phases in CP and DA participants receiving MMA+ compared with corresponding CP and DA groups receiving MMA−, and will not differ between CF groups receiving MMA+ or MMA.

### Secondary End Points

#### Hypothesis 2a

CP and DA participants randomized to MMA+ will report significant pre-post increases in levels of present moment awareness and state mindfulness compared with participants randomized to MMA−, and present moment awareness and state mindfulness will not differ between CF groups receiving MMA+ or MMA.

#### Hypothesis 2b

CP, DA, and CF participants receiving MMA+ will report significantly lower mind wandering than corresponding participants receiving MMA−, reflecting the potential for MMA+ to make participants more able to recover their attention from mind-wandering contents and processes during MM.

#### Hypothesis 3

CP and DA participants randomized to the MMA+ condition will demonstrate significant reductions in mood symptoms (state depression and anxiety) after the MM task compared with CP and DA participants randomized to the MMA− condition, reflecting the potential for MMA+ to facilitate the emotion regulation effects of MM for clinical populations.

## Methods

### Design

**Figure 2:**
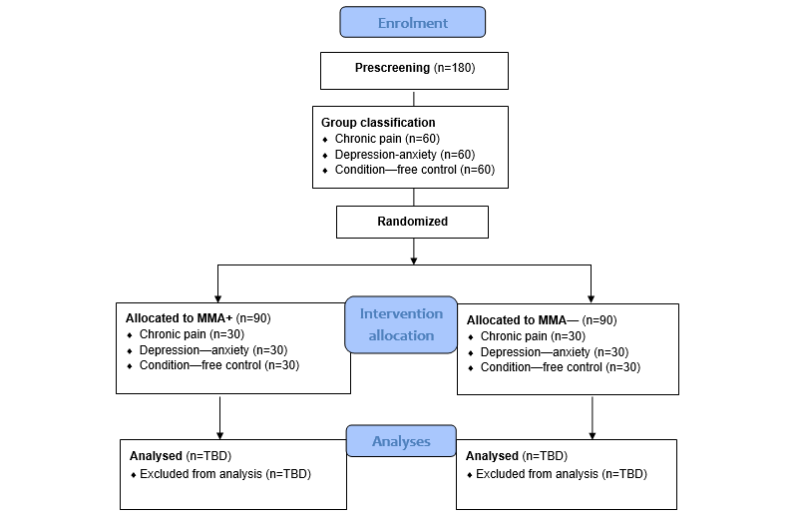
Consolidated Standards of Reporting Trials 2010 flow diagram showing anticipated participant flow for enrolment, group classification, randomization to intervention, and analyses. MMA−: mindful breathing practice without using the Mindfulness Meditation app; MMA+: mindful breathing practice using the Mindfulness Meditation app; TBD: to be determined.

This RCT study was designed according to the 2010 Consolidated Standards of Reporting Trials statement (Multimedia Appendix 1) [30], reviewed and approved by the York University research ethics board (Human Participants Review Committee protocol number e2017-303), and registered with ClinicalTrials.gov (NCT03296007) on September 22, 2017, before the recruitment of the first participant. A total of 180 male and female participants will be recruited from the Web-based York Undergraduate Research Participant Pool, based on voluntary participation, and classified into 3 groups of approximately 60 participants each (and randomized into MMA+ or MMA−) based on their prescreening questionnaires: CP (n=30 MMA+ and n=30 MMA−), depression-anxiety (n=30 MMA+ and n=30 MMA−), and CF participants (n=30 MMA+ and n=30 MMA−; Figure 2).

### Sample Size Estimation

Sample size estimate for a repeated-measures linear mixed-effects analysis of variance (ANOVA) with 3 groups (CP, DA, and CF), 2 conditions (MMA+, MMA−), and 3 phases (baseline, stress induction, and MM) indicates that a total of 141 participants are needed to detect small-to-medium effect size (f=0.15) HRV changes with a type I error rate (alpha) of .05, a power of 0.95, and a 0.50 correlation between repeated measures (G*Power; Heinrich Heine University Düsseldorf). The small-to-medium effect size corresponds to previously published studies of HRV increases during brief mindfulness practice [13,14]. Recruitment of more than 180 participants will allow for an attrition rate of approximately 20% because of withdrawals, dropouts, technical failures, and missing data. On the basis of prescreening and recruitment numbers in our past pilot study (VV Latman, MA, unpublished data, June 2017), we found the percentage of participants reporting diagnosed CP, severe depression, or anxiety symptoms to be 27%, 33%, and 41%, respectively. Accordingly, the combined number of students expected to be prescreened for eligibility to enroll 60 participants per group will be approximately N=550.

### Inclusion and Exclusion Criteria

Participants will be eligible for the study if they are enrolled in a course at York University that provides course participation credit via study enrolment through the Undergraduate Research Participant Pool website. On the recruitment website as well as on the consent form, we have explained that the study may require use of a smartphone-based task. Participants with self-reported cardiac conditions (eg, cardiac arrhythmias, coronary artery disease, and pacemaker) will be excluded as they contravene the interpretation of vagal-mediated HRV [31]. Participants in this study will have no prior relationship with the researchers, including participation in classes by faculty members on the research team.

### Procedures

#### Group Classification and Randomization

Upon signing up for the study, participants will be instructed to arrive to the Human Pain Mechanisms Lab at York University, where the study procedures will be explained, and informed consent will be obtained from all participants. Participants will complete prescreening questionnaires for CP, depression, or anxiety symptoms. A research assistant will score the questionnaire once completed and check to see if the participant meets *severe* criteria for depression symptoms (≥21 on the Centre for Epidemiological Studies Depression Scale) [32] or anxiety symptoms (≥36 on the Beck Anxiety Inventory) [33]. If they meet such criteria, they will be classified into the depression-anxiety group and randomized to the MMA (MMA+) or no app (MMA−) condition. If the participant reports having a CP condition (>3 months of pain on the Brief Pain Inventory) [34], they will be classified into the CP group and randomized to MMA+ or MMA−. Participants who do not meet the criteria for CP or severe depression-anxiety symptoms will be classified into the CF group and randomized to MMA+ or MMA−.

A 3-block randomization schedule (CP, depression-anxiety, and condition free) with 2 treatment arm allocations (MMA+ and MMA−) was created by the study coinvestigator (study author MAA) using a randomization sequence generator [35]. The randomization schedule uses a 1:1 ratio with blocks of 10. Treatment allocation will be determined by study coordinators and staff members after participants complete their prescreening questionnaires to determine their group classification (Figure 2). Study research assistants review prescreening questionnaires and assign the grouped participants to their corresponding treatment arm using a randomization schedule column concealed in a computer spreadsheet using the *highlight* (black) function. Upon entering participants into the spreadsheet, research assistants unhighlight the next available cell in the corresponding column to reveal their treatment assignment.

#### Experimental Session

**Figure 3:**
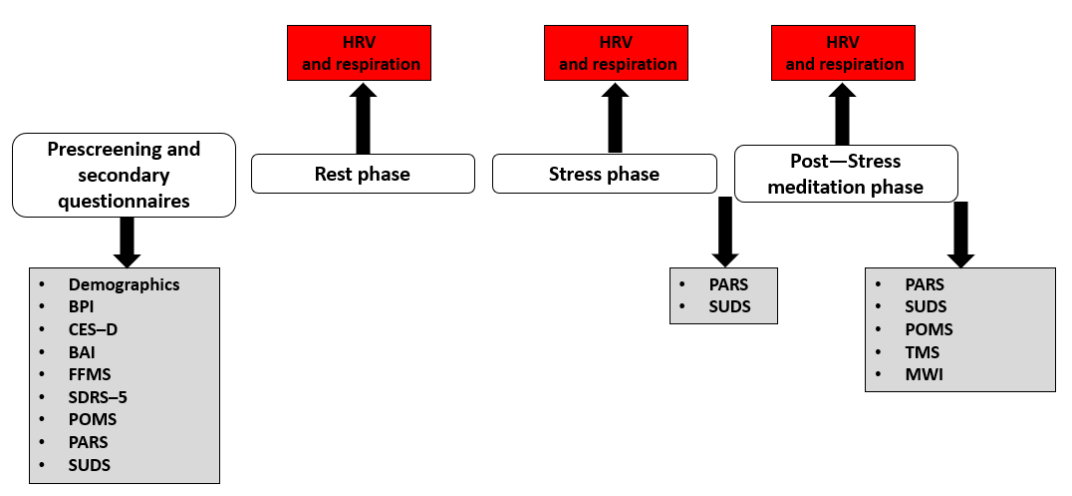
Study procedures. Physiological assessments are in red boxes, and questionnaire assessments are in gray boxes. BAI: Beck Anxiety Inventory; BPI: Brief Pain Inventory; CES-D: Center for Epidemiology Depression Scale; FFMS: Five-Factor Mindfulness Scale; HRV: heart rate variability; MWI: Mind-Wandering Inventory; PARS: Present Moment Awareness Rating Scale; POMS: Profile of Mood States; SDRS-5: Social Desirability Rating Scale-5; SUDS: Subjective Units of Distress Scale. TMS: Toronto Mindfulness Scale.

Participants will undergo a 3-phase (baseline, stress induction, and MM) assessment during which electrocardiogram (ECG) and respiration rate data will be collected for later HRV analysis (Figure 3). A mobile cardiogram system by MindWare (MindWare Technologies, LTD) will be used, requiring placement of 3 adhesive electrodes on the right clavicle and left and right hipbones and a respiratory belt secured around the participant’s waist.

##### Rest Phase

The first phase will comprise a 5-min baseline period during which participants will rest quietly with their eyes closed and not speak or make sudden movements.

##### Stress Induction Phase

Following the baseline phase, a 5-min stress induction procedure will be performed whereby the participant engages in a set of mental arithmetic tasks, with the instruction to work as quickly as possible for maximal performance. Mental arithmetic was chosen as a stressor task because of its superior properties in eliciting sympathetic responses compared with other laboratory-based stressors [36].

##### Post-Stress Meditation Phase

After the stress induction phase, a post-stress MM phase will involve either the MMA+ or MMA− task according to the randomization schedule.

After completing the prestudy set of questionnaires, participants will be randomized to either the smartphone-based MMA+ or a mindful breathing practice without use of a mobile app (MMA−). Mindful breathing instructions will involve paying attention to breathing sensations while seated with eyes closed for 12 min and reorienting attention back to breathing once aware of mind wandering [2]. Research assistants remain in the room during the intervention phase, for both intervention conditions, to ensure there are no technical or practical issues with the interventions. There are no adverse events expected during the study, and participants are free to withdraw their participation at any time during the study with no penalty to their research credit or any other costs.

### Intervention Arm: Mindfulness Meditation With the App

The MMA was developed using JavaScript and installed on an iPhone 4s. As the app was designed with young adults in mind, the leading thought was to keep the intervention brief and the design simple and easy to use. Participants are asked to hover their thumbs on 2 buttons, *breath* and *other*, presented on a white screen for the duration of the task (Figure 1). The MMA is a 12-min breath awareness task that involves a total of 24 silent phases comprising 6 different durations (5 seconds, 15 seconds, 25 seconds, 35 seconds, 45 seconds, and 55 seconds) randomly presented 4 times each and followed by the presentation of a 1-second tone. During the silent phases, participants are to practice mindful breathing with their eyes closed by paying attention to their breathing sensations and reorienting their attention when noticing their mind wandering. At the sound of the tone, participants press *breath* if, in that moment, they were attending to their breath, or *other* if they were attending to other experiences. Participants are instructed to return their attention to mindful breathing after pressing 1 of the 2 buttons on the screen. The app was designed to record reaction time from tone onset to button press and the number of times *breath* and *other* buttons were pressed. A prototype was first tested internally, and feedback was gathered from the research team and pilot participants to ensure a smooth user experience without technical bugs. Feedback influenced decisions regarding the number of tones and interval between tones played, the size of the *breath* and *other* buttons, and the addition of a 1-min trial period before commencing the 12-min meditation task.

Participants in the MMA+ condition will use the MMA to practice mindful breathing with the following instructions: “For the duration of the task, pay attention to your breathing sensations, including (1) the feeling of the air passing through your nostrils, (2) the movement of your in-and-out breath at your chest and torso, (3) the sound of the air as you breathe in and out, (4) the temperature (coolness or warmth) of the in-and-out breath, and (5) returning your attention to your breath when you have noticed your attention has been elsewhere.” In addition, amidst mindful breathing practice, they will be asked to press a *breath* (I am paying attention to my breath) or *other* (I am not paying attention to my breath) button on the phone screen when hearing a tone introduced at random intervals. The MMA task will involve a total of 24 silent phases of 6 different durations (5 seconds, 15 seconds, 25 seconds, 35 seconds, 45 seconds, and 55 seconds) randomly presented 4 times each and followed by the presentation of the tone. Participants are provided a 1-min practice session with the app before the intervention session. Participants will engage in smartphone-based mindful breathing for 12 min in a seated and eyes closed position.

### Control Arm: Mindfulness Meditation With No App

Participants in the MMA− condition will engage in mindful breathing without use of the smartphone app for 12 min in a seated and eyes closed position.

### Measures

#### Heart Rate Variability

There are several metrics that quantify HRV, the majority of which are either time based or frequency based. With the beat-to-beat interval series (also referred to as the R-R series), one can estimate the square root of the mean of squared successive differences between interbeat intervals (IBI), which has been found to be strongly correlated with respiration-based heart rate (HR) changes) [37]. It is important to note that both the IBI and the standard deviation of IBI’s are subject to increases with longer ECG recordings (influenced by slower, long-range fluctuations in HR independent of respiration-induced beat-to-beat changes). Thus, the Task Force [38] has recommended 5 min as a standardized length of HRV assessment in clinical and psychophysiological research. Using the frequency domain measure, one can examine the extent to which the HR varies within specific frequency ranges. The Fourier transform method deconstructs the time domain representation of the R-R series and computes a measure of *power* in several frequency bands in units of milliseconds squared (ms^2^). These bands include ultralow frequency (<0.04 Hz), low frequency (0.04-0.15 Hz), and high frequency (HF; 0.15-0.4 Hz). Typically, the frequency band of interest for the purposes of HRV interpretation is the HF band, as this is where the respiratory-linked beat-to-beat changes are reflected [37,39]. Although this is not intended to be an exhaustive account of HRV metrics, the aforementioned measures are the most commonly used in studies of vagal influence on cardiac chronotropy. On the basis of the recommendations by the 2 international committees [38,40], this study focuses on the frequency measure as the primary measure to interpret changes in vagal-mediated HRV across different conditions.

ECG recordings will be collected using MindWare Impedance Cardiograph acquisition system and used to analyze HF-HRV as the primary HRV measure. The MindWare system utilizes 3 adhesive electrodes applied to the right collarbone (negative lead) and the lower left and right ribs (positive lead and ground lead) or alternatively the wrists (positive and negative leads) and ankle (ground lead) if necessary. MindWare BioLab and HRV software will be used to calculate time- and frequency-based HRV metrics. Patients will be measured during phases of (1) rest in a seated position with eyes closed (5 min), (2) a stress induction task requiring rapid completion of arithmetic problems (5 min), and (3) meditation conditions of MMA+ or MMA−.

#### Respiration Rate

A MindWare respiratory belt (below the sternum) will be used to monitor respiration rate during rest, stress induction, and meditation. Respiration is a potential confounding variable for HRV interpretation and will be used as a covariate in HRV analyses if it is found to differ between assessment phases or study groups [31].

#### Breath Focus and Reaction Time

Using data collected with the MMA+, breath focus will be measured using the ratio of *breath* and *other* responses during the MMA+ task. Reaction time measures will also be collected pertaining to mean (seconds) in pressing *breath* and *other* buttons during the MMA+ task.

#### Prescreening Questionnaires

The following prescreening questionnaires will be administered to students to determine group classification by assessing for clinical CP (The Brief Pain Inventory), depression (The Center for Epidemiological Studies-Depression Scale), and anxiety symptoms (Beck Anxiety Inventory).

##### Brief Pain Inventory—Short Form

The Brief Pain Inventory is a 16-item, self-report questionnaire that measures pain intensity and pain interference. The test has good internal consistency (alpha=.85) and high test-retest reliability [41].

##### Center for Epidemiological Studies Depression Scale

The Center for Epidemiological Studies Depression Scale (CES-D) is a screening test for depressive symptoms with good sensitivity and specificity and high internal consistency [32]. The CES-D has been found acceptable and reliable in adolescent and young adult populations [42].

##### Beck Anxiety Inventory

The Beck Anxiety Inventory is a 21-question multiple-choice self-report questionnaire that is used for measuring anxiety severity. Internal consistency (Cronbach alpha) ranges from .92 to .94 for adults and test-retest (1-week interval) reliability is 0.75 [33].

#### Secondary Outcomes and Trait Questionnaires

Participants will also be asked to complete the following questionnaires to assess mood (Profile of Mood States [POMS]), present moment awareness (Present Moment Awareness Ratings Scale [PMARS]), mind-wandering (Mind-Wandering Inventory [MWI]), state mindfulness (Toronto Mindfulness Scale [TMS]), trait mindfulness (Five Factor Mindfulness Scale), social desirability (Social Desirability Response Set 5-Item Survey), and subjective stress (Subjective Units of Distress Scale [SUDS]).

##### Profile of Mood States

The POMS is a 37-item questionnaire designed to assess global distress as well as 6 mood states: fatigue, vigor-activity, tension-anxiety, depression, anger-hostility, and confusion-bewilderment. Participants are asked to indicate the degree to which they have experienced different mood states in the past week. The scale has good internal consistency (alpha=.91) and test-retest reliability (*r*=0.74). The POMS has been validated for use in adolescents and adults [43]. Higher total scores represent greater total mood disturbance [44].

##### Present Moment Awareness Ratings Scale

The PMARS is a 5-item questionnaire developed specifically for use in this study. Participants will be asked to rate their level of awareness of different aspects of present moment experiences. The PMARS was used in a previous pilot study (VV Latman, MA, unpublished data, June 2017), and statistical analyses of its psychometric properties is underway [45].

##### Mind-Wandering Inventory

The MWI is a 5-point Likert-based questionnaire developed by the authors with items retroactively assessing the frequency of different types of mind-wandering events the respondent experienced during the mindful breathing task. The MWI was used in a previous pilot study (VV Latman, MA, unpublished data, June 2017), and statistical analyses of its psychometric properties is underway [46].

##### Toronto Mindfulness Scale

The TMS is a state measure of mindfulness with 2 factors: curiosity and decentering. The TMS defines curiosity as an awareness of present moment experience with a curious attitude. Decentering is defined as an awareness qualified by distance and separation from current experience. The TMS has good internal consistency of alpha=.95. Current research demonstrates that the TMS is a reliable and valid measure of mindfulness that accurately measures curiosity and decentering [47].

##### Five-Factor Mindfulness Scale

The Five-Factor Mindfulness Scale (FFMS) is a 39-item Likert-based scale that assesses 5 aspects of mindfulness: nonreactivity to inner experience, acting with awareness, describing, nonjudging of inner experience, and observing. Research has demonstrated the FFMS to be valid in community and student samples with an internal consistency of alpha greater than .90 [48].

##### Socially Desirable Response Set 5-Item Survey

The Socially Desirable Response Set 5-Item Survey (SDRS-5) is a self-report measure designed to assess the tendency for individuals to provide socially desirable responses to self-reports of attitudes, behaviors, and feelings. Alpha reliability estimates for this instrument have been found to be between .66 and .68 [49].

##### Subjective Units of Distress Scale

The SUDS is a means of rating the severity of current distress (or anxiety), allowing for the monitoring of changes over time, where 0 is feeling perfectly relaxed and 100 is the worst anxiety and stress imaginable [50]. Participants will be asked to provide 0 to 100 SUDS ratings at baseline, post-stress induction, and after the post-stress meditation phases. The SUDS ratings will be used to do a manipulation check with respect to the mental arithmetic stressor and to monitor changes in stress over time.

### Statistical Analyses of Outcomes

This study has 2 coprimary hypotheses based on published recommendations for HRV analyses [31,39]. Generally, within-subject designs and analyses are preferred considering the high interindividual variations observed in HRV measures. To analyze differences in HRV with respect to MMA+ and MMA− between groups, change scores from stress to MM will be computed by subtracting stress-HRV from MM-HRV and used as the dependent variable in hypothesis 1a. In addition, within-subject changes in HRV according to the MM conditions and groups will be examined in hypothesis 1b. Results will be interpreted using significance values, confidence intervals, and effect sizes [51]. Intention-to-treat methodology will be the guiding principle of analyses. A complete case analysis approach will be used in anticipation of missing or unusable data.

#### Hypothesis 1a

To analyze HRV change in groups between the stress and MM phases, a linear mixed model will be used with group (CP, DA, and C) and condition (MMA+ and MMA−), with HRV change scores from stress to MM phases as the dependent variable. Planned comparisons will be computed comparing the MMA+ and MMA− conditions for each group (CP, DA, and C).

#### Hypothesis 1b

To analyze HRV differences between groups and across phases, a 3-way linear mixed-effects ANOVA will be used with group (CP, DA, and C), phase (baseline, stress, and MM), and condition (MMA− and MMA+), with HRV as the dependent variable. Significant interactions will be followed up with simple effects analyses comparing the stress and MM phases for each group (CP, DA, C) within the 2 conditions (MMA+ and MMA−).

#### Hypotheses 2a and 2b

To analyze pre-post increases in levels of present moment awareness, state mindfulness, and mind wandering, separate 3-way repeated-measures ANOVA with group (CP, DA, and C), time (pre and post), and condition (MMA− and MMA+) will be used with simple main effects to examine significant interactions.

#### Hypothesis 3

To analyze pre-post changes in mood, a 3-way ANOVA with group (CP, DA, and C), time (pre and post), and condition (MMA− and MMA+) will be used with simple main effects to examine significant interactions.

### Exploratory Analyses

Associations between HRV and psychometric measures will be explored with Pearson correlations and linear regression models using the following constructs: present moment awareness (PMARS), mind wandering (MWI), anxiety symptoms (Anxiety Sensitivity Index), depressive symptoms (CES-D), state mindfulness (TMS), and mindfulness skills (FFMS). Breath focus and reaction time will be examined for group differences (CP, DA, and C) using 1-way ANOVA. Given potential response biases in self-reported outcomes, we will examine group differences in socially desirable responding (SDRS-5) using 1-way ANOVA and use it as a control variable in applicable outcome analyses.

## Results

Recruitment for the study began in November 2017 and is expected to be completed in winter of 2019-2020. Data collection is currently underway. As of July 2019, we have recruited 189 total participants (C*P*=41, DA=55, and CF=93). Data analysis, manuscript writing, and additional publications are expected to be completed in the fall and winter of 2019.

## Discussion

### Principal Findings

The main findings of the study are expected to reveal a positive pattern of HRV responses across the different study groups, CP, DA, and C, such that they exhibit significantly increased HRV (*P*<.05) in the treatment condition, MMA+, in comparison with the control condition, MMA−. On the basis of previous research published by our team, HRV is expected to increase during MM when practiced after stressor tasks [13,14]. However, a pattern of *inflexible* HRV responses has been noted in previous studies involving participants with clinical characteristics. For instance, a randomized experimental study of students high on perfectionistic traits and nonperfectionist students was conducted with measurement of HRV at baseline, stress, and during subsequent audio-guided MM or a rest condition with an audio lecture. Only the nonperfectionist group exhibited significantly increased HRV in the mindfulness condition, whereas the perfectionists did not, reflecting an inflexible state of sympathetic dominance [13]. A follow-up study was conducted with headache and headache-free participants where both groups showed increased HRV during post-stress mindfulness. However, during post-stress rest, the headache group exhibited significantly lower HRV compared with headache-free participants, reflecting impaired ability for cardio-vagal recovery [14]. This pattern of results is consistent with literature indicating low and inflexible HRV patterns in individuals with clinical characteristics of depression, anxiety, and chronic pain [52-54].

The MMA is designed as a supportive training tool to aid clinical populations with mindful breathing practice. Accordingly, our primary hypothesis is that HRV will increase from the stress to MM phases in DA and CP participants randomized to MMA+ compared with MMA−. Specifically, mindful breathing practice with the aid of the MMA is expected to yield additional relaxation effects for participants, by way of respiratory sinus arrhythmia, to be reflected in increased HRV measures. Examination of secondary hypotheses will help to further contextualize the role of key psychological processes, including present moment awareness, state mindfulness, mind wandering, and mood, as mediators and outcomes of mindfulness-based treatments.

### Limitations

A potential limitation of this study is the lack of blinding of research assistants with respect to group screening and treatment allocation procedures. Research assistants are required to remain in the room to address any technical or practical issues with the intervention, negating the possibility of blinding research assistants to treatment. However, both MMA+ and MMA− conditions entail app- or self-guided mindful breathing practice with no active involvement of research assistants; thus, risk of bias in intervention delivery is low. Another limitation is that the use of the MMA presents a demand characteristic requiring participants to reliably report *breath* or *other* responses. Any accidental responses by participants that do not correspond to their attentional states (eg, pressing *breath* when the intention was to press *other*) cannot be corrected during the MMA task, and may momentarily distract participants during the task. In addition, participants will only be provided 1 session of the MMA task, which is a limited duration of exposure that prevents examination of dose-response effects. In future longitudinal studies, we hope to test repeated practice with the app and its effects on HRV, pain, mood, and other outcomes. In terms of study groups, classification criteria did not exclude individuals who reported mild-to-moderate levels of depressive-anxiety symptoms or pain for less than 3 months, instead classifying subcriteria participants as condition free. The presence of symptoms in the CF group has the potential to impact results. With regard to study outcomes, it must be noted that the MWI and present moment awareness rating scales are awaiting psychometric validation and will undergo factor analyses based upon the data gathered in this and other studies. One procedural limitation is that the MMA− condition is not structurally identical to the MMA+ condition as it foregoes the use of a smartphone app during the practice of mindful breathing. To preserve task similarity, it may be useful in future studies of the MMA to provide participants with a *control app* that does not provide random-interval tones as they practice mindful breathing. Finally, the study is based on undergraduate student populations, limiting generalizability until future studies are conducted with community and clinical populations.

### Conclusions

This RCT will contribute to the burgeoning health psychology literature about the clinical relevance of HRV measures in the assessment and treatment of psychiatric and health conditions. The findings may also contribute to the growing use of HRV as a biomarker and biofeedback tool within clinical and health psychology. Furthermore, there is an evolving need for an evidence basis related to supportive mindfulness training tools for the self-management of symptoms related to depression, anxiety, and chronic pain. The innovation of incorporating mindful breathing practice into a scalable app provides valuable information to allow for future iterative development of an app involving novel techniques for teaching mindfulness to clinical populations.

## Appendix

Multimedia Appendix 1 CONSORT-EHEALTH checklist (V 1.6.1).

## Abbreviations

ANOVA: analysis of variance
ANS: autonomic nervous system
CF: condition-free
CES-D: Center for Epidemiological Studies Depression Scale
CP: chronic pain
DA: depression or anxiety
ECG: electrocardiogram
FFMS: Five-Factor Mindfulness Scale
HF: high frequency
HR: heart rate
HRV: heart rate variability
IBI: interbeat intervals
MM: mindfulness meditation
MMA: Mindfulness Meditation app
MMA−: mindful breathing practice without using the Mindfulness Meditation app
MMA+: mindful breathing practice using the Mindfulness Meditation app
MWI: Mind-Wandering Inventory
PMARS: Present Moment Awareness Ratings Scale
POMS: Profile of Mood States
RCT: randomized controlled trial
SDRS-5: Socially Desirable Response Set 5-Item Survey
SUDS: Subjective Units of Distress Scale
TMS: Toronto Mindfulness Scale
